# Non-Invasive *In Vivo* Imaging of Tumor-Associated CD133/Prominin

**DOI:** 10.1371/journal.pone.0015605

**Published:** 2010-12-20

**Authors:** Chizuko Tsurumi, Norbert Esser, Elke Firat, Simone Gaedicke, Marie Follo, Martin Behe, Ursula Elsässer-Beile, Anca-Ligia Grosu, Ralph Graeser, Gabriele Niedermann

**Affiliations:** 1 Department of Radiation Oncology, University Hospital Freiburg, Freiburg, Germany; 2 Department of Internal Medicine I, University Hospital Freiburg, Freiburg, Germany; 3 Department of Nuclear Medicine, University Hospital Freiburg, Freiburg, Germany; 4 Department of Urology, University Hospital Freiburg, Freiburg, Germany; 5 ProQinase GmbH, Freiburg, Germany; National Cancer Institute, United States of America

## Abstract

**Background:**

Cancer stem cells are thought to play a pivotal role in tumor maintenance, metastasis, tumor therapy resistance and relapse. Hence, the development of methods for non-invasive *in vivo* detection of cancer stem cells is of great importance.

**Methodology/Principal Findings:**

Here, we describe successful *in vivo* detection of CD133/prominin, a cancer stem cell surface marker for a variety of tumor entities. The CD133-specific monoclonal antibody AC133.1 was used for quantitative fluorescence-based optical imaging of mouse xenograft models based on isogenic pairs of CD133 positive and negative cell lines. A first set consisted of wild-type U251 glioblastoma cells, which do not express CD133, and lentivirally transduced CD133-overexpressing U251 cells. A second set made use of HCT116 colon carcinoma cells, which uniformly express CD133 at levels comparable to primary glioblastoma stem cells, and a CD133-negative HCT116 derivative. Not surprisingly, visualization and quantification of CD133 in overexpressing U251 xenografts was successful; more importantly, however, significant differences were also found in matched HCT116 xenograft pairs, despite the lower CD133 expression levels. The binding of i.v.-injected AC133.1 antibodies to CD133 positive, but not negative, tumor cells isolated from xenografts was confirmed by flow cytometry.

**Conclusions/Significance:**

Taken together, our results show that non-invasive antibody-based *in vivo* imaging of tumor-associated CD133 is feasible and that CD133 antibody-based tumor targeting is efficient. This should facilitate developing clinically applicable cancer stem cell imaging methods and CD133 antibody-based therapeutics.

## Introduction

Many malignant tumors contain a subset of so-called cancer stem cells (CSCs) with features similar to those of normal stem cells. The frequencies of CSCs are found to vary from less than 1 to more than 25% of the cells in a tumor [1]–[3]. Cardinal features of these stem-like cells are long-term self-renewal, tumorigenicity upon xenotransplantation to immunocompromised mice, and a certain differentiation capacity. Thus, xenotransplanted tumors initiated by CSCs tend to reflect the cellular heterogeneity of the original tumors. In contrast, more differentiated primary tumor cells are less or completely non-tumorigenic in immunocompromised mice [1], [2]. There is growing evidence that CSCs drive metastasis [1], [4], [5]. Moreover, they seem to be highly resistant to chemotherapy and radiation and may thus be crucial in tumor recurrence after conventional therapy. Resistance mechanisms discussed include the expression of multidrug resistance transporters, slow cycling, efficient repair of DNA damage and a high expression of antiapoptotic proteins [6], [7].

CSCs express stem cell-specific transcription factors such as Sox2 as well as other intracellular stem- and progenitor cell markers such as nestin, musashi, or aldehyde dehydrogenase [8]. More important for the identification of live CSCs, CSC-specific surface markers have also been identified and major efforts are ongoing to identify new markers. For example, human breast CSCs appear to express a surface marker combination of lin-/CD44hi/CD24low/-/ESA+ [8]. Another CSC marker found in a multitude of tumor entities is CD133/prominin. Prominin-1 (CD133) was originally described in mouse embryos as a marker for neuroepithelial progenitor cells [9], and in humans as the AC133 antigen, a marker of hematopoietic stem and progenitor cells recognized by the monoclonal antibody (mAb) AC133 [10]. CD133/prominin is a highly glycosylated transmembrane protein. Stem cells are characterized by a set of glycosylation-dependent epitopes in the extracellular portion of CD133 recognized by the mAb AC133 (CD133/1) and the mAbs AC141 and 293C (CD133/2). These epitopes are lost upon differentiation [11]. Meanwhile, CD133/prominin (AC133) has been identified as a CSC marker for brain tumors including glioblastoma, ependymoma and medulloblastoma, as well as for a variety of non-nervous system tumors such as pancreatic, colon, bronchial, prostate, ovarian and liver cancer, melanoma, and leukemia [12], [13]. In gliomas, the frequency of cells expressing CD133 (AC133) increases with tumor grade [14]. CD133 (AC133)-positive CSCs of a variety of tumor entities have been shown to be particularly chemo- and radioresistant [4],[15],[16]. Thus as a putative CSC marker for a wide variety of tumor entities, CD133 (AC133) is one of the most intensively investigated of the known CSC markers.

In view of their above described essential role, non-invasive imaging of CSCs would be of great value in the management of malignant diseases; e.g., for determining prognosis, monitoring therapeutic efficacy and influencing therapeutic protocols [17]. For noninvasive imaging of CSCs as well as CSC-specific therapies, the targeting of cell surface proteins using antibodies or other receptor ligands is particularly relevant [18]. Although there are some recent reports of therapeutic targeting of CSC marker-expressing tumor cells with mAbs [19]–[21], non-invasive *in vivo* imaging of unmanipulated CSCs or CSC-marker expressing tumor cells has not been reported thus far [17], [22].

Our long-term goal is to develop antibody-based probes and methods for the noninvasive imaging of CSCs *in vivo*, specifically of CD133+ CSCs. However, CSCs may often constitute only a small population of the cells in a tumor. Thus there is a need for imaging modalities and probes that provide high sensitivity and high image resolution [17]. Here, as a first step towards this goal, we assessed whether it is possible to detect CD133 expressed on xenograft tumors by noninvasive antibody-based *in vivo* imaging. For this purpose, we used isogenic pairs of CD133 positive and negative tumor cell lines as robust and reproducible tumor models with defined intrinsic negative control. As an antibody, we employed the mAb AC133, which is commercially available and is widely used for enrichment and isolation of CD133+ CSCs [13]. Near-infrared fluorescence molecular tomography (FMT), a highly sensitive and fully quantitative technology, was employed as the *in vivo* imaging modality [23], [24].

## Materials and Methods

### Cell lines

HCT116 and HCT116 p53−/− colon carcinoma cells [25], as well as the parental U251 glioma cells (ATCC, Manassas, VA, USA) and a L1-CD133 lentivirus transduced CD133-overexpressing U251 cell pool were cultured in DMEM (Invitrogen, Darmstadt, Germany) with 10% FCS (PAA laboratories, Pasching, Austria) supplemented with 1% penicillin/streptomycin (PAA laboratories), 1x MEM non-essential amino acids (Invitrogen) and 2 mM L-glutamate (PAA laboratories). The primary glioblastoma stem-like cells were generated from a glioblastoma sample of a patient with primary glioblastoma multiforme (Firat *et al*., Delayed cell death associated with mitotic catastrophe in γ-irradiated stem-like glioma cells. PLoS ONE, in revision) and were cultured in Neurobasal-A medium (Invitrogen) supplemented with 20 ng/ml epidermal growth factor and 20 ng/ml fibroblast growth factor (from Prospec, Rehovot, Israel), 1% penicillin/streptomycin (PAA laboratories), 0.5x MEM non-essential amino acids, 1x GlutaMax-I (Invitrogen) and B27 supplement without vitamin A (Invitrogen).

### Plasmids

The human CD133 full-length cDNA (IRAUp969F1267D) was purchased from imaGenes (Berlin, Germany), and digested with EcoRI. The CD133 ORF was then inserted into the EcoRI site of the lentiviral vector L1-puro, which is a derivative of the 433_L1_myc_IRES_mitov#A65EA_cm5 [26] in which the GFP was replaced by a puromycin resistance gene and the myc gene was removed to create the L1-puro-CD133 lentiviral vector.

### Lentiviral transductions

293T cells were co-transfected with the L1-puro-CD133 plasmid, pMD2.G (Addgene, Cambridge, MA, USA), which encodes the VSV-G surface gene, and the pCMV8.74 vector (Addgene), which provides the env and gag genes, using the calcium phosphate method in the presence of 20 µM chloroquine (Sigma-Aldrich, Seelze, Germany). Eight hours after transfection, the medium was replaced by fresh DMEM. Lentiviral supernatants were harvested to transduce U251 cells in the presence of 5 µg/ml polybrene (Sigma-Aldrich) 36 and 60 h after transfection. The transduced cells were selected using 1 µg/ml puromycin.

### Antibodies

Anti-CD133/1 (AC133.1)-phycoerythrin (PE), anti-CD133/2 (293C3)-PE, and CD133/1 (W6B3C1) [10] were from Miltenyi Biotec (Bergisch Gladbach, Germany), anti-mouse PE-conjugated F(ab')_2_-fragments from Dianova (Hamburg, Germany), and PE-Cy7-conjugated anti-mouse CD11b from NatuTec (Frankfurt am Main, Germany).

### Antibody purification

The AC133.1 hybridoma cells (ATCC HB-12346) were cultured in DMEM with Ultra low IgG FBS (Invitrogen). The supernatant of a 250 ml culture was applied on a 5 ml protein G-Sepharose column (GE Healthcare, München, Germany) and the Sepharose was washed with PBS. The antibody was then eluted using 100 mM glycine (pH 2.5), and the eluate immediately neutralized using 1 M Tris-HCl (pH 8.8). The eluted antibody was concentrated with Amicon Ultracel-50 k concentrators (Millipore, Billerica, MA, USA) and dialysed against PBS. The antibody concentration was determined by measuring the absorbance at 280 nm using a NanoDrop ND-100 spectrophotometer (Peqlab, Erlangen, Germany).

### Antibody labeling

Antibodies were labeled and purified using the Alexa-488 protein- or the Alexa-647 IgG-labeling kit (Invitrogen) according to manufacturer's protocols. Briefly, 1 mg AC133 antibody was brought to a pH higher than 8.3 by adding 100 mM sodium bicarbonate solution to a final volume of 0.5 ml. The antibody solution was transferred to a vial of Alexa Fluor 488 reactive dye and incubated at room temperature for 1 h in the dark. Labeled antibodies were purified from free dye by gel filtration using a column provided in the kit. Cy5.5 labeling was performed using CyDye Cy5.5 mono-reactive NHS esters (GE Healthcare). A dye/protein ratio of 2 to 3 dye molecules per antibody was first determined by mixing 100 µg purified AC133 antibody with a serial dilution of CyDye Cy5.5 mono-reactive NHS esters. For large scale labeling, 1 mg purified AC133 antibody was incubated with 60 µM CyDye Cy5.5 mono-reactive NHS ester in 100 mM disodium hydrogen phosphate (pH 9) for 2 h. Labeled antibodies were purified using a PD10 column (GE Healthcare).

### Antibody titration and flow cytometry

Serially diluted antibody was incubated with 5×10^5^ CD133-overexpressing U251 or HCT116 wild-type cells suspended in 100 µl of wash buffer (0.5% BSA/2 mM EDTA in PBS) for 15 min. After washing, the cells were incubated with 1.5 µg of an anti-mouse PE-conjugated F(ab')_2_-fragment in 100 µl wash buffer for 20 min. Samples were then washed twice and assayed on a Cytomix FC-500 flow cytometer (Beckman Coulter, Krefeld, Germany). Statistical analyses were performed using Graphpad Prism 4.0b statistical software (GraphPad Software Inc., San Diego, CA, USA).

### Immunofluorescence microscopy

1×10^6^ HCT116 or HCT116 p53−/− cells were incubated in wash buffer (see above) with 5 µg/ml AC133-Alexa647 antibody for 20 min at 4°C. After washing with wash buffer, the cells were fixed with 4% paraformaldehyde, and mounted onto slides in Aqua PolyMount (Polysciences, Inc., Warrington, PA, USA) supplemented with 1 µg/ml 4,6-diamidino-2-phenylindole (DAPI; Roche, Basel, Switzerland). Pictures were taken using an Olympus ScanR fluorescence microscope (Olympus, Hamburg, Germany).

### Western blotting

Cells were lysed in RIPA buffer (1% NP40, 1% sodium deoxycholate, 0.1% SDS, 0.15 M NaCl, 0.01 M sodium phosphate [pH 7.2], 2 mM EDTA, 50 mM sodium fluoride, 0.2 M sodium orthovanadate) supplemented with the protease inhibitor cocktail ‘Complete’ from Roche and proteins were quantified using a BCA assay (Thermo Scientific, Bonn, Germany). 40 µg lysate were applied to a 10% SDS gel and blotted onto a Hybond-ECL membrane (GE Healthcare). CD133 antibody at a concentration of 0.25 µg/ml was incubated for 3 h and detected using a peroxidase-conjugated F(ab')_2_-fragment goat anti-mouse (Dianova). Actin antibody was incubated at a concentration of 0.25 µg/ml for 1 h and detected using a peroxidase-conjugated F(ab')_2_-fragment goat anti-rabbit (Dianova). The blots were visualized by ECL (GE Healthcare).

### Animal experiments

All animal experiments were performed in accordance with the German Animal License Regulations and were approved by the animal care committee of the Regierungspräsidium Freiburg (registration number: G-10/64). Subcutaneous (s.c.) xenografts were induced in nude (NMRI-nu/nu) mice (Charles River, Sulzfeld, Germany). 5×10^6^ U251 wild-type, CD133-overexpressing U251, or HCT116 wild-type cells or 4×10^6^ HCT116 p53−/− cells were injected per animal in 100 µl PBS. When the size of the tumor reached 200–300 mm^3^, 75 µg Cy5.5-labeled antibodies and 2 nmol Integrisense 750 (PerkinElmer/Visen Medical, Bedford, MA, USA) were injected via the tail vein, and the fluorescence signal was visualized using a FMT1500 Fluorescence Molecular Tomography system (PerkinElmer/VisEn Medical) at day 1, 2, 3, 4, and 7 for the HCT116 xenografts and at day 1, 2, 4, and 7 for the U251 xenografts; 9 days after dye injection all mice were euthanized by cervical dislocation and the isolated tumors were scanned with the FMT system. For the determination of the lowest number of HCT116 wild-type cells detectable by FMT, increasing percentages (0, 20, 40, 60, 80, 100%) of HCT116 wild-type cells were mixed with HCT116 p53−/− cells and stained with the AC133-Cy5.5 antibody in wash buffer (see above) for 30 min at 4°C. After washing, 2×10^6^ cells were resuspended in 25 µl PBS, mixed with an equal volume of matrigel, and injected s.c. into the flank of nude mice. *In vivo* fluorescence signals were visualized using a FMT1500 within 1–2 h after tumor cell injection. After the experiment, all mice were euthanized by cervical dislocation.

### *In vivo* FMT imaging

Mice were anesthetized by gas anesthesia (isoflurane/oxygen mixture), before placing them into an imaging cassette (PerkinElmer/VisEn Medical). After positioning the cassette in the FMT1500 imaging system (PerkinElmer/VisEn Medical), reflectance images were captured in white light and fluorescence (2D planar). For 3D imaging, a field around the tumor was selected, and the tomographic scan was carried out. The scan data were analyzed using reconstruction software provided by the manufacturer (PerkinElmer/VisEn Medical). For tomographic data analysis, three-dimensional regions of interest (ROI) were drawn around the tumor, and the total amount (in picomoles) of fluorochrome was calculated by TrueQuant software (PerkinElmer/VisEn Medical) using previously generated standards of the appropriate dye.

### Pre-tests for tumor digestion experiments

3×10^6^ HCT116 wild-type or HCT116 p53−/− cells grown in 10 cm dishes were incubated with 4 µg/ml AC133-Alexa488 antibody overnight. Cells were then washed with PBS and digested with 0.7 U/ml Liberase-Blendzyme (Roche, Basel, Switzerland)/Accutase (eBioscience, Frankfurt, Germany) for 50 min at 37°C. After washing with 0.5% BSA/2 mM EDTA in PBS, the cells were stained with 5 µg/ml of anti-CD133/2 (293C3)-PE and analyzed by flow cytometry.

### Tumor digestion

Tumor pieces were digested with 0.7 U/ml Liberase-Blendzyme (Roche)/Accutase (eBioscience)/100 U DNaseI (Invitrogen)/10 mM MgCl_2_ for 20 min at 37°C. Non-digested pieces were further digested with accutase for 30 min. Red blood cells were removed using RBC Lysis Buffer (eBioscience). Cells were then washed with PBS, and suspended in wash buffer or a 2.4G2 hybridoma supernatant as an Fc blocker (for the CD11b staining only). After 10 min, 5 µg/ml of anti-CD133/2 (293C3)-PE or 10 µg/ml of PE-Cy7-conjugated anti-mouse CD11b antibody was added, and the cells were analyzed by flow cytometry.

### Statistical Analysis

Results are presented as mean ± SD. Data were compared using the unpaired 2-tailed Student's t test. A p-value <0.05 was considered significant (*: p<0.05 and **: p<0.01). Analyses were performed using GraphPad Prism software version 4.0b (GraphPad Software Inc., San Diego, USA).

## Results

**Figure 1:**
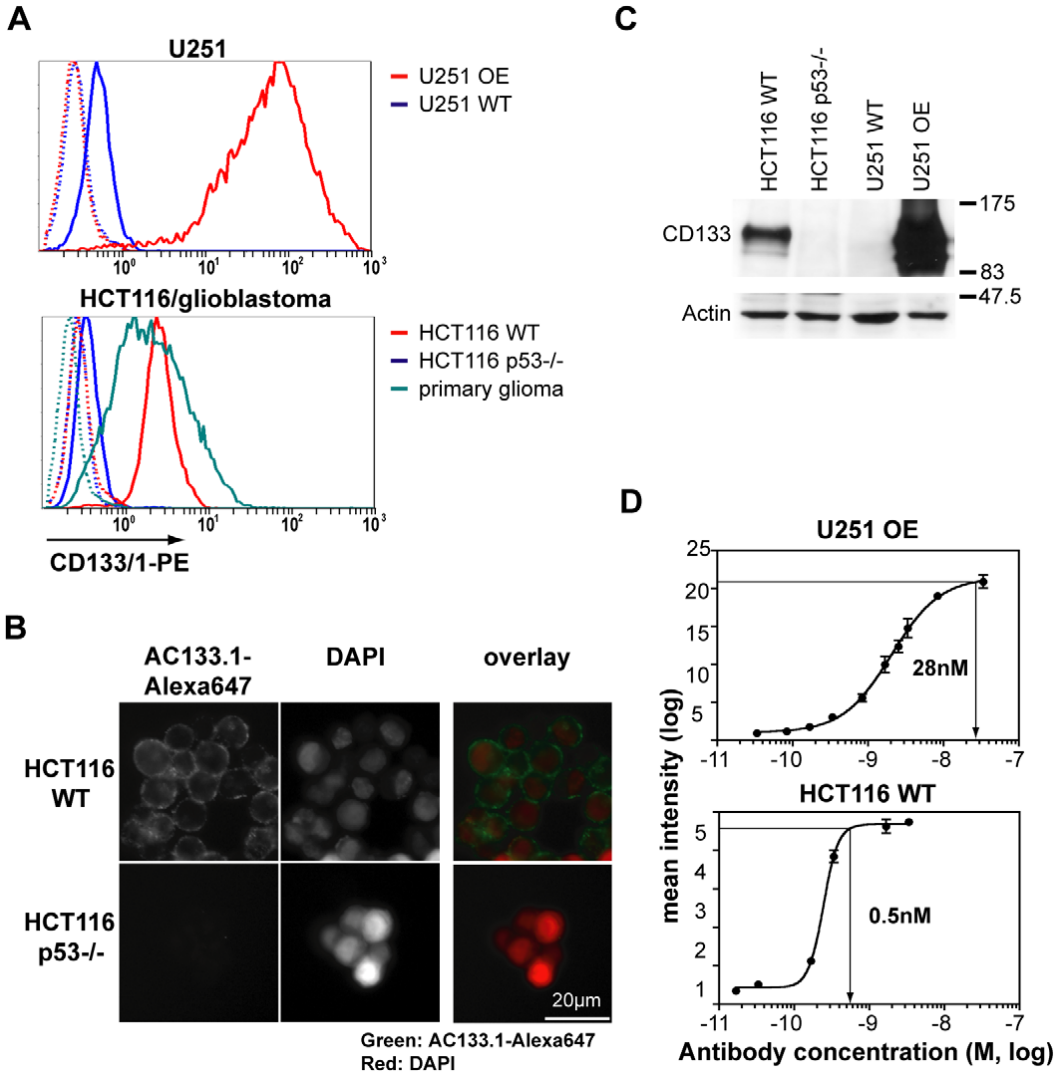
Characterization of cell lines and the AC133.1 antibody. (A) CD133 expression of HCT116, U251, and primary glioblastoma stem-like cells as determined by flow cytometry. Upper panel: U251 wild-type cells and CD133-overexpressing U251 cell lines; lower panel: HCT116 wild-type, HCT116 p53−/−, and primary glioblastoma stem-like cells. Cells were incubated with the CD133/1-PE antibody and analyzed by flow cytometry. The dotted lines show the unstained controls. (B) CD133 expression of HCT116 wild-type and HCT116 p53−/− cell lines as determined by immunofluorescence microscopy. After incubation with the AC133-Alexa647 antibody, cells were counterstained with DAPI. (C) Levels of total cellular CD133 protein as determined by Western blotting. Actin served as a loading control. (D) Determination of the saturation concentration of AC133.1 antibody for HCT116 wild-type or CD133-overexpressing U251 cells via flow cytometry. The cells were stained using serial dilutions of the purified antibody and analyzed by flow cytometry using an anti-mouse PE-conjugated F(ab')_2_ fragment. The saturation concentrations were determined using prism software. WT, wild-type; OE, overexpressing.

The AC133.1 mAb, which recognizes CD133 on tumor stem cells, was chosen as a probe to detect CD133 expressing cells *in vivo*. Besides a specific detection tool, a robust mouse model system was required in order to develop a sensitive detection protocol. In analogy to previously reported *in vivo* imaging studies, e.g., monitoring the epidermal growth factor receptor family [27], we decided to generate a CD133-overexpressing cell line. To this end, the CD133-negative U251 glioblastoma cell line was transduced with a lentiviral construct encoding the full-length CD133 cDNA. Flow cytometry analysis of these cells using the fluorescently labeled AC133.1 antibody demonstrated an increase of antigen expression by >100 fold compared to wild-type cells (Figure 1A, *upper panel*). An isogenic CD133+/− pair of HCT116 cell lines served as an additional model system. Wild-type HCT116 colon carcinoma cells uniformly express CD133 [28] at a level similar to that found on primary glioblastoma stem-like cells. In stark contrast, HCT116 p53−/− cells [25] were found to be virtually CD133 negative (Figure 1A, *lower panel*). The flow cytometry data were verified using immunostaining and a Western blot analysis (Figures 1B and C).

The relative surface CD133 expression levels of the positive cell lines were determined measuring their AC133.1 antibody saturation concentration via flow cytometry. The saturation point for AC133.1 on CD133-overexpressing U251 cells was found to be 2.8×10^−8^ M, and on HCT116 cells 5×10^−10^ M, establishing the CD133 expression level difference between HCT116 wild-type cells and the CD133-overexpressing U251 cell line at ca. sixtyfold (Figure 1D). Hence, the pair of wild-type and CD133-overexpressing U251 glioma cells was initially tested as a model system for CD133 *in vivo* imaging.

**Figure 2:**
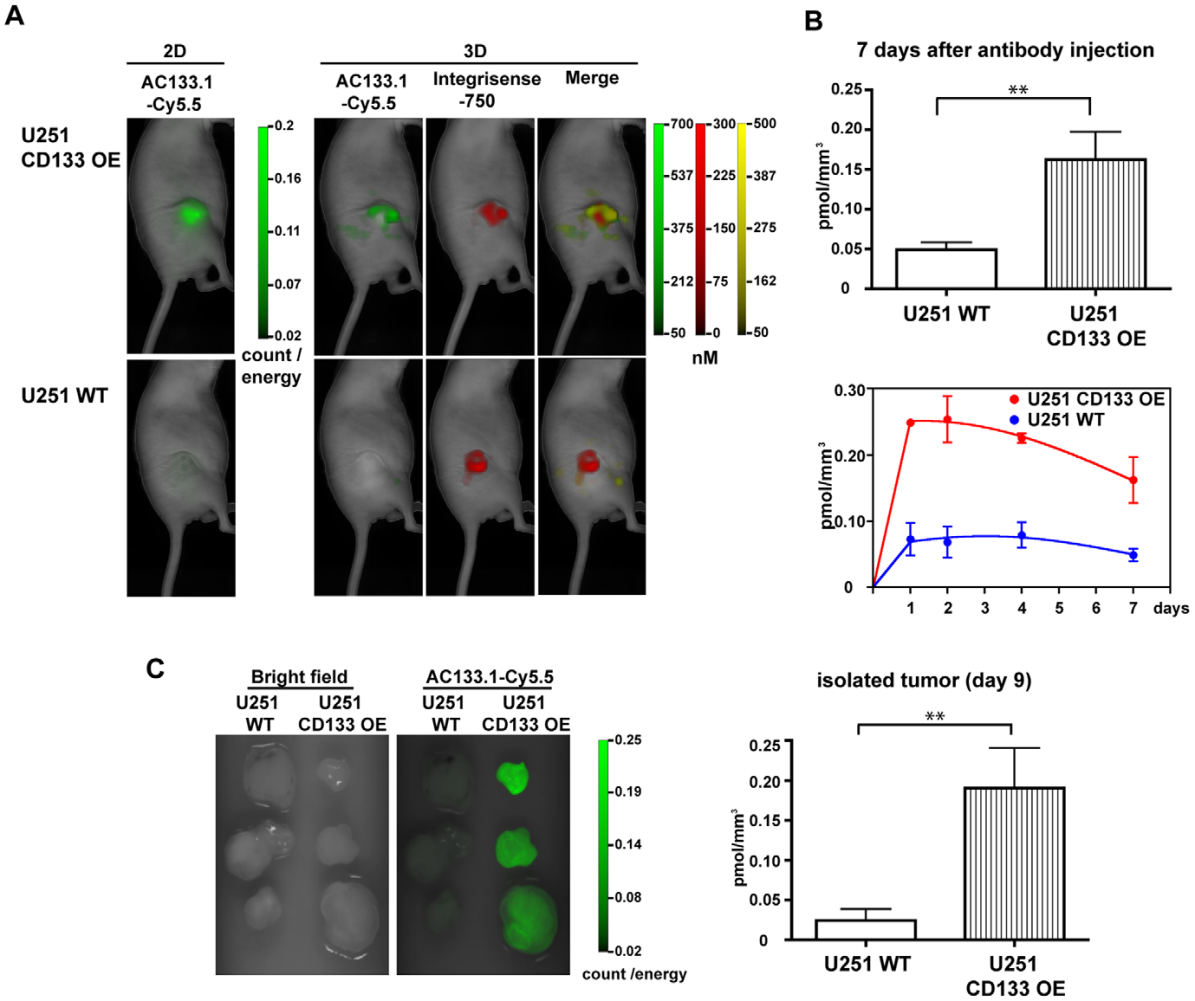
*In vivo* imaging of U251 xenografts in mice injected with Cy5.5-labeled AC133.1 antibody. (A) U251 s.c. xenograft mice [U251 wild-type cells (lower panel) and CD133-overexpressing U251 cells (upper panel)] were injected with 75 µg AC133.1-Cy5.5 and 2 nmol of IntegriSense 750. One, 2, 4 and 7 days later, the mice were imaged using the VisEn FMT-1500 Fluorescence Molecular Tomography system. The pictures presented correspond to the last measurement at day 7. (B) Quantification of AC133.1-Cy5.5 accumulation in U251 wild-type and CD133-overexpressing U251 xenografts. Signal intensity (pmol/mm^3^) of the xenografts 7 days after AC133.1-Cy5.5 antibody injection (upper panel), and the resulting time course over 7 days (lower panel). (C) Signal intensity of isolated tumors 9 days after AC133.1-Cy5.5 injection. Tumors were resected from mice and imaged with an FMT-1500. WT, wild-type; OE, overexpressing.

In order to test whether the AC133.1 antibody could detect CD133 expression *in vivo*, CD133-overexpressing and wild-type U251 cells were subcutaneously (s.c.) implanted into nude mice (for a tumor growth curve, see Figure S1). At a tumor size of 200 to 300 mm^3^, 75 µg of Cy5.5-labeled AC133.1-antibody was injected into the tail vein of each mouse, along with 2 nmol IntegriSense 750, and the resulting *in vivo* fluorescence signal was detected using the FMT1500 after 1, 2, 4 and 7 days. IntegriSense 750 served as an independent fluorescence marker to localize the tumor; it is an optimized *in vivo* fluorescence imaging agent that targets integrin alpha v beta 3, a key biomarker highly expressed on actively angiogenic endothelium and certain types of tumor cells [29]. A marked difference in AC133.1-Cy5.5-mediated fluorescence signal intensities between wild-type and CD133-overexpressing U251 tumors was observed in the reflectance image (2D) as well as the tomographic image (3D) (Figure 2A). A quantification of the signals revealed a more than threefold higher AC133.1 antibody concentration in the CD133-overexpressing U251 tumors compared to the control tumors 7 days after antibody injection (Figure 2B). The relative signal intensities between CD133-overexpressing and negative cells remained fairly constant over the course of the experiment, except that, toward the end, the signal from the positive tumors dropped faster than that of the control (Figure 2B). Scanning of the isolated tumors 9 days after dye injection confirmed the *in vivo* results (Figure 2C).

**Figure 3:**
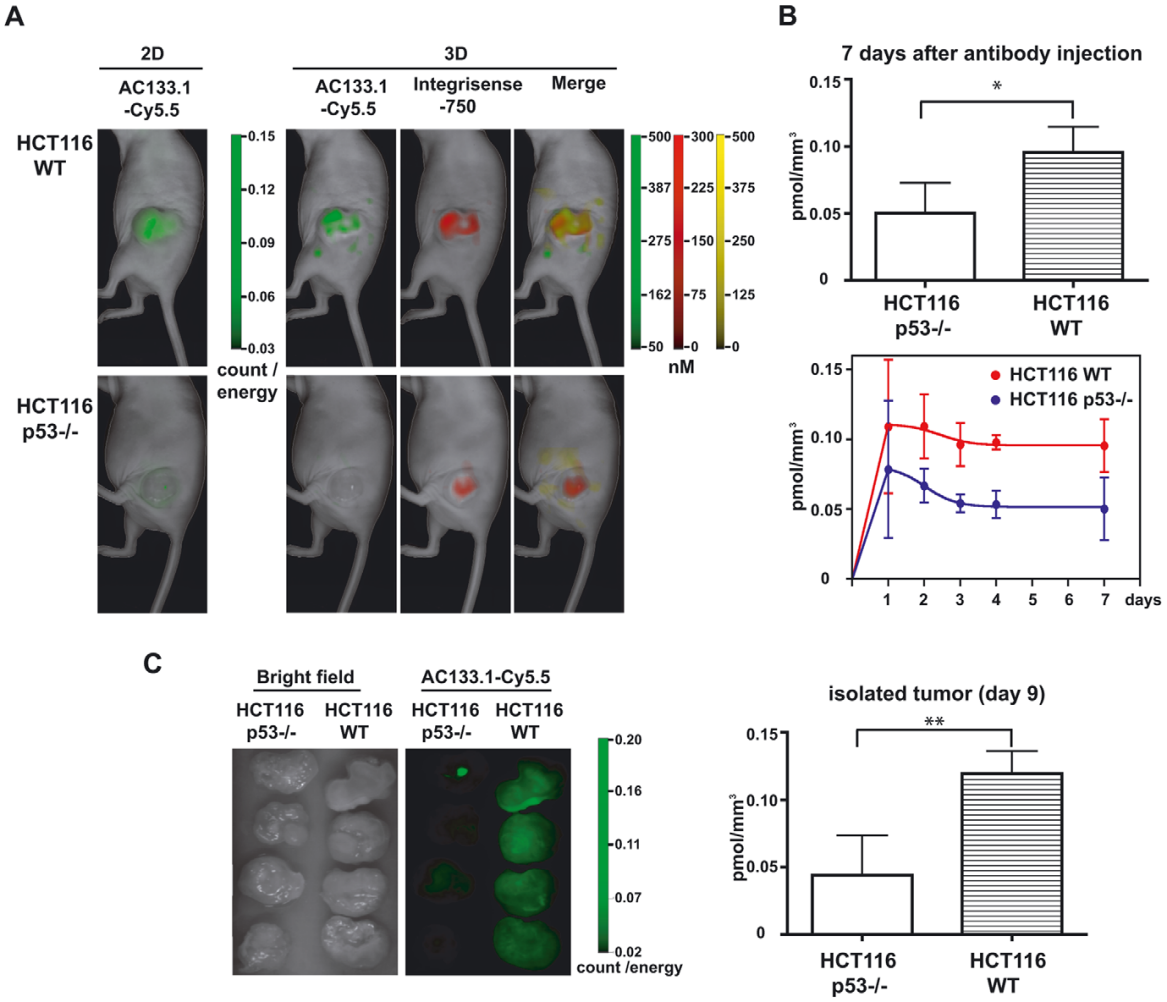
*In vivo* imaging of HCT116 xenografts in mice injected with Cy5.5-labeled AC133.1. (A) HCT116 s.c. xenograft mice [HCT116 wild-type cells (upper panel) and HCT116 p53−/− cells (lower panel)] were injected with 75 µg AC133-Cy5.5 and 2 nmol of IntegriSense 750. One, 2, 3, 4 and 7 days later, the mice were imaged using the FMT-1500 system. The pictures presented correspond to the last measurement at day 7. (B) Quantification of AC133.1-Cy5.5 accumulation in HCT116 and HCT116 p53−/− xenografts. Comparison of signal intensities (pmol/mm^3^) between the xenografts at day 7 after AC133.1-Cy5.5 injection (upper panel) and the time course over 7 days (lower panel). (C) Signal intensity of isolated tumors 9 days after AC133.1-Cy5.5 injection. Tumors were resected from mice and imaged with an FMT-1500 system. WT, wild-type.

Since imaging worked well using an overexpressing cell line, we next tested whether the antibody could detect s.c. implanted xenografts of HCT116 cells, which express CD133 at an endogenous level. To this end, CD133-positive HCT116 wild-type or CD133-negative HCT116 p53−/− cells were implanted into nude mice (for a tumor growth curve, see Figure S1), and similar experiments were performed as described above. Again, the fluorescence intensities between the two cell lines differed markedly, whether reflectance image (2D) or tomographic imaging (3D) was used to analyze the signals (Figure 3A). A twofold higher concentration of the AC133.1 antibody was detected in the HCT116 wild-type tumors than in the CD133-negative HCT116 p53−/− tumors, 7 days after injection of the antibody (Figure 3B). The relative signal intensities remained more or less constant over the course of the experiment, with significant differences between positive and negative tumors. Only at the first measurement 24 h after antibody injection significant differences could not be detected. The *in vivo* results could again be confirmed via imaging of the isolated tumors (Figure 3C).

**Figure 4:**
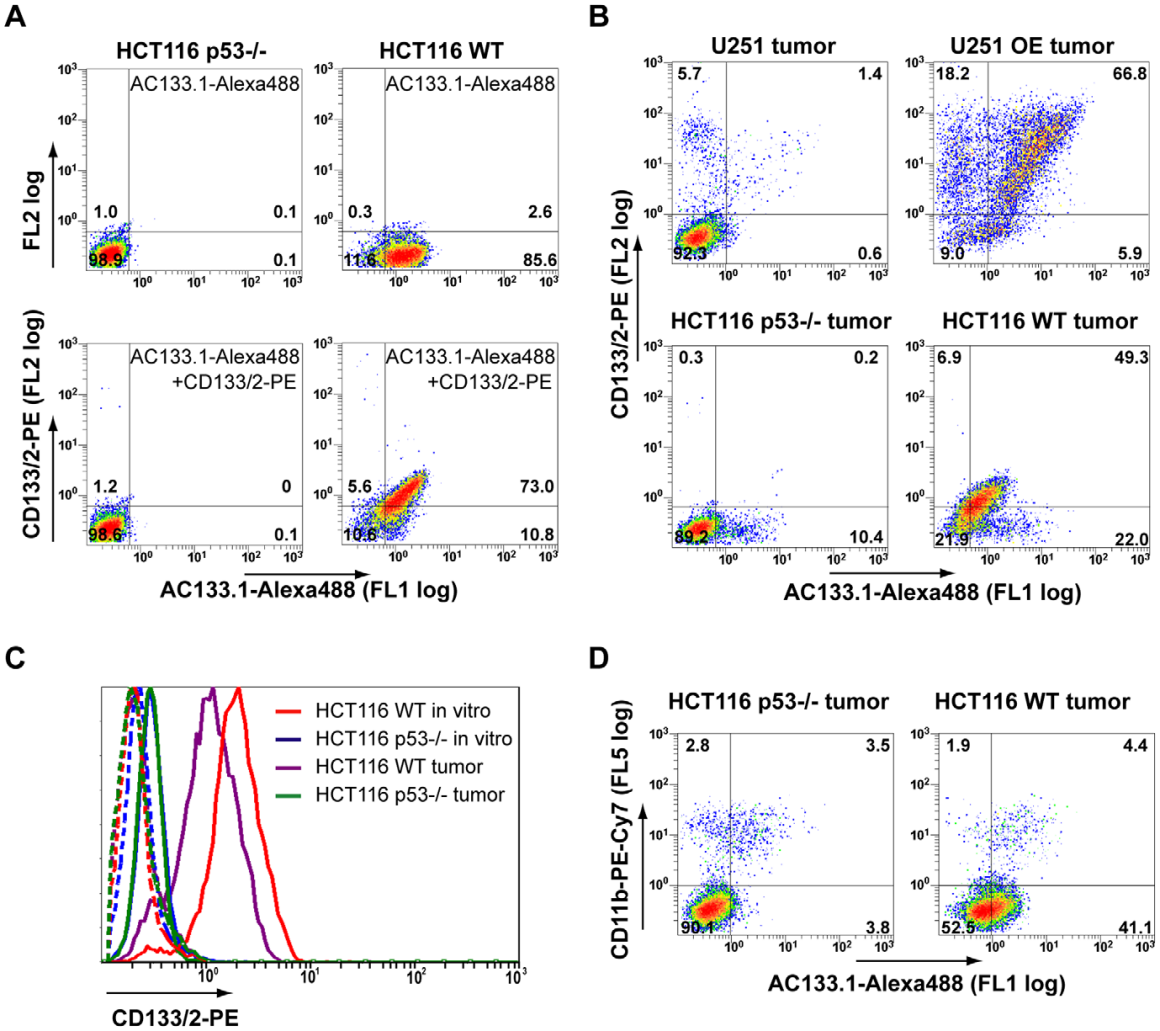
Flow cytometric detection of i.v.-injected AC133.1-Alexa488 antibody on cells isolated from tumors. (A) Pre-test for tumor digestion experiments showing that CD133 and prebound antibody can be detected after blendzyme/accutase digestion. HCT116 wild-type and HCT116 p53−/− cells were incubated with AC133.1-Alexa488 overnight and digested with blendzyme/accutase for 50 min. The treated cells were then stained with CD133/2-PE antibody and analyzed by flow cytometry. (B) Blendzyme/accutase digested tumors of AC133-Alexa488 injected mice were stained with CD133/2-PE antibody and analyzed by flow cytometry. (C) Downregulation of CD133 by s.c. growing HCT116 wild-type cells. Cells from digested tumors and *in vitro* cultured HCT116 cells were stained with CD133/2-PE antibody and CD133 expression levels were determined by flow cytometry. The dotted lines show the unstained controls. (D) Tumor macrophages contribute to the signal of *in vivo* injected AC133-Alexa488. Digested tumors were incubated with anti-mouse Fc-receptor blocking antibodies, stained thereafter with PE-Cy7-CD11b antibody and analyzed by flow cytometry. In Figures 4A, B, and D, the percentages of cells falling into the negative, single- and double-positive quadrants are shown. The quadrants were set according to the autofluorescence of the cells (for the gating controls and for statistical analyses, see Figures S2, S3, S4, and S5). Due to the lower CD133 expression and lower antibody binding *in vivo* compared to *in vitro*, the percentage of the cells in the different quadrants is difficult to precisely determine for HCT116 wild-type tumors but the shift towards the double-positive quadrant of the majority of cells can clearly be seen (lower right panel in B; see also Figure S4). FL, fluorescence; WT, wild-type; OE, overexpressing.

Although the signal differences between CD133-negative and -positive tumors were statistically significant for both pairs of cell lines used, the binding of the injected antibodies to their target still had to be confirmed. Also, compared to the *in vitro* flow cytometry results, the differences between CD133-negative and -positive cells were far less pronounced *in vivo*, particularly in the case of the U251 cells. Insufficient antibody injected, or an incomplete penetration of the tumors by the antibody are possible explanations for this apparent reduction of the dynamic range of the signals. In order to address these issues, tumors were excised 3 days after the injection of the Alexa488 labeled AC133 antibody and single-cell suspensions were prepared. The single cell suspensions were stained with a mAb recognizing another epitope of CD133 (CD133/2) [10], to obtain a measure of the total available CD133 molecules, and analyzed by flow cytometry. Previously performed control experiments confirmed that the treatment of HCT116 cells with accutase and blendzyme, the enzymes used for preparing single-cell suspensions, did not remove cell-surface CD133 nor bound AC133.1-Alexa488 antibodies (Figure 4A).

In single-cell suspensions of CD133-overexpressing U251 tumors, besides a large double positive population, cells positive only for the *in vitro* added CD133/2 antibody were also detected, indicating that the amount of injected antibody had not been sufficient (Figure 4B, *upper right panel*). In contrast, in similar preparations from CD133-positive HCT116 wild-type tumors essentially all cells were double positive, suggesting that the injected antibody had fully occupied the available CD133 epitopes *in vivo*. However, the flow cytometry signals for both CD133 antibodies were rather low (Figure 4B, lower right panel).

A decrease of the CD133 expression levels *in vivo* might provide an explanation for the apparently smaller difference in CD133 expression *in vivo* compared to *in vitro*. Indeed, when compared to *in vitro* cultured cells, HCT116 wild-type cells isolated from xenografts exhibited lower CD133 expression (Figure 4C).

We also found that a small population of CD133-negative cells in the tumors bound injected AC133.1-Alexa488. In order to determine whether these represent Fc-receptor-positive cells such as macrophages, which bind antibodies via their Fc-portion, single-cell suspensions were stained with the macrophage marker CD11b. As shown in Figure 4D, a significant population of CD11b-positive cells had indeed bound injected AC133.1 (double-positive population in the left and right panel of Figure 4D).

Finally, the *in vivo* detection limit for HCT116 wild-type cells was determined. For this purpose, CD133-positive HCT116 wild-type and CD133-negative HCT116 p53−/− cells were mixed at ratios ranging from 0–100% wild-type cells, labeled with AC133.1-Cy5.5 antibody *in vitro*, and injected s.c. into nude mice. As shown in Figure S6, 1.6×10^6^ wild-type HCT116 cells in a 80% wild-type (CD133+) to 20% p53−/− (CD133-) cell mix could clearly be detected with the FMT1500.

## Discussion

We present here results on the specific accumulation of a fluorescently labeled AC133.1 mAb in CD133-positive xenograft tumors detected by non-invasive *in vivo* fluorescence imaging. These data provide the first evidence that non-invasive *in vivo* imaging of tumor-associated CD133 (AC133), a well-known CSC-marker, is feasible.

Relative to their negative counterparts, substantially higher amounts of injected antibody were found on both CD133-overexpressing U251 and HCT116 wild-type cells, the latter expressing CD133 at endogenous levels similar to those of primary CD133+ glioblastoma stem-like cells. The lower CD133 expression level of the HCT116 wild-type tumors led to a generally lower signal to noise ratio compared to CD133-overexpressing U251 tumors. Thus, the difference in the AC133.1-Cy5.5-mediated fluorescence signal between CD133-positive and -negative HCT116 tumors reached statistical significance only after 48 hours, probably when injected antibodies had been cleared from blood circulation. This slow ‘background clearing’ is often observed in antibody-mediated *in vivo* imaging and is related to the high molecular weight as well as the main clearance route via the liver [30]. The lower signal to noise ratio of the HCT116 system was further reduced by downregulation of CD133 expression *in vivo* and non-specific antibody binding to tumor stroma macrophages. In the digested cell mix derived from the HCT116 wild-type xenografts, macrophages with bound AC133 antibody constituted less than 10% of antibody-bearing cells. However, macrophages can represent > 50% of the cells in a tumor [31]. Although preventing Fc receptor binding might help to reduce the background signals, the benefit of using small antibody fragments lacking the Fc portion like Fab, F(ab)_2_, or single chain constructs for *in vivo* imaging is contradictory [32]. Reasons are a lower affinity in the case of monovalent fragments and lower tumor uptake due to their fast excretion. The opposite is true for imaging with mAbs, which have a very slow blood clearance rate. High target affinity, sufficient tumor uptake due to better pharmacokinetics and reduced background due to diminished Fc receptor binding may be achieved by engineering recombinant antibodies or relatively large antibody fragments with a mutated Fc portion [33].

Another problem encountered during our studies is the decrease of CD133 expression in HCT116 tumor xenografts. A similar observation has already been described for a melanoma cell line [34]. Cells with CD133 expression lower than that of HCT116 wild-type cells may be hard to identify unequivocally with this imaging method unless a proper negative control is available. Moreover, tumor stem cells may represent only a small fraction of the whole tumor; e.g., in glioblastomas, CD133 (AC133)-positive cells range between 0 and 50% of the total cells [35]. With our current method, 1.6×10^6^ cells in a mix containing 80% CD133-positive wild-type cells could be detected. However, the detection of CSCs in real tumors may be further complicated by the way these cells are distributed within the tumor. Whereas a fraction of CSCs may be concentrated in certain tumor regions (e.g., hypoxic regions) or may occur in clusters [14], many CSCs are probably spread throughout the tumor. Reliably imaging very low numbers of scattered cells is very challenging. It requires probes providing an exceptionally good signal-to-noise ratio, i.e., high-affinity probes with fast tumor penetration, sufficient circulation time, and low unspecific binding.

A homogeneous tumor penetration of the imaging probes is also important since this is required to obtain reliable information on the spatial distribution of CSCs within the tumor. Such topological information would be important to optimize treatment planning, e.g., in radiation therapy higher irradiation doses could be applied to particularly resistant tumor subregions such as hypoxic or CSC-rich regions by so-called ‘dose painting’ [36]. Homogeneous tumor penetration would also be important for therapeutic targeting of CSCs. Our study demonstrates that the AC133.1 antibody readily finds its antigen in tumor xenografts *in vivo*, so it might be worthwhile to derivatize the antibody with toxins [19], or use the antibody to target liposomally encapsulated drugs.

Any potential diagnostic and therapeutic use of CD133-specific antibodies in humans would require further investigations on the expression pattern and level of CD133 epitopes in normal tissues and the degree of specificity/cross-reactivities of available CD133-specific mAbs. The AC133 and AC141 epitopes are expressed on normal tissue stem and progenitor cells such as hematopoietic stem and endothelial progenitor cells [10], [37]. However, neural stem- and progenitor cells in the stem cell-rich regions of the adult human brain appear to be negative for these CD133 epitopes [38]. The epitopes seem to be expressed also on a subset of normal tissue epithelial cells, but expression on normal tissue adjacent to tumors has been described as being substantially lower than in the malignant tumors studied [39]. If targeting CD133 (AC133) should not prove specific enough for the detection of CSCs *in situ* [40], possible solutions might include the identification of more specific CSC-targets or the production of dual specificity recombinant antibodies, i.e., with one arm specific for CD133 and the other for another CSC-antigen.

Taken together, our data provide first evidence that noninvasive *in vivo* imaging of tumor-associated CD133 (AC133) is feasible. In addition, they show that antibody-based targeting of CD133 (AC133) is efficient. However, additional efforts are still necessary to characterize additional CSC markers, and to further develop imaging probes and imaging modalities before a small population of CSCs may be monitored reliably and noninvasively *in vivo*.

## Supporting Information

Figure S1 **Growth curves for U251 and HCT116 xenograft tumors.** Nude mice were injected s.c. with 5×10^6^ cells with the exception of HCT116 p53−/− cells which were injected at a number of 4×10^6^ to adjust the growth rate to the HCT116 wild-type tumors. Tumor sizes were measured twice weekly with a caliper. Individual tumor volumes were calculated by the formula V =  [length × (width)^2^]/2. WT, wild-type; OE, CD133 overexpressing
(TIF)

Figure S2 **Autofluorescence of the cells used for the flow cytometric analyses presented in** **Figure 4A** **.** The percentages of cells falling into the negative, single- and double-positive quadrants are shown. FL, fluorescence; WT, wild-type
(TIF)

Figure S3 **Controls and statistics for the flow cytometric analyses of the U251 tumors presented in** **Figure 4B** **.** The upper panels demonstrate the gating according to the autofluorescence. Upper left subpanels: Autofluorescence of tumor cells from ‘control mice’ that had not been injected with AC133.1-Alexa488 antibody. Lower left subpanels: Fluorescence of tumor cells from non-injected ‘control mice’ stained with a PE-labeled CD133/2-specific antibody *in vitro*. Upper right subpanels: Fluorescence of tumor cells from mice that had been injected with AC133.1-Alexa488 antibody. Lower right subpanels: Fluorescence of tumor cells from injected mice stained with a PE-labeled CD133/2-specific antibody *in vitro* as shown in Figure 4B. The lower two panels show a statistical analysis of the percentages of tumor cells falling into the four quadrants (n = 3). FL, fluorescence; WT, wild-type; OE, overexpressing
(TIF)

Figure S4 **Controls and statistics for the flow cytometric analyses of the HCT116 tumors presented in** **Figure 4B** **.** The upper panels demonstrate the gating according to the autofluorescence. Upper left subpanels: Autofluorescence of tumor cells from ‘control mice’ that had not been injected with AC133.1-Alexa488 antibody. Lower left subpanels: Fluorescence of tumor cells from non-injected ‘control mice’ stained with a PE-labeled CD133/2-specific antibody *in vitro*. Upper right subpanels: Fluorescence of tumor cells from mice that had been injected with AC133.1-Alexa488 antibody. Lower right subpanels: Fluorescence of tumor cells from injected mice stained with a PE-labeled CD133/2-specific antibody *in vitro* as shown in Figure 4B. The percentages of cells falling into the negative, single- and double-positive quadrants are shown. The middle and the lower panels show biological comparison controls (HCT116 wild-type vs. HCT116 p53−/− xenograft cells). Middle panel: The red and the black circled regions include the main cell population of a wild-type and a p53−/− xenograft, respectively. These populations do not overlap, demonstrating that the majority of the CD133+ cells in the wild-type xenografts had bound the injected AC133.1-Alexa488 antibody. Lower right panel: Statistical analysis of the mean fluorescence intensities for the Alexa488 and the PE signal (n = 6 wild-type and 5 p53−/− tumors, respectively). The cell populations analyzed fell into the gates shown in the lower left panel. FL, fluorescence; MFI, mean fluorescence intensity; WT, wild-type; OE, overexpressing
(TIF)

Figure S5 **Controls and statistics for the flow cytometric analyses presented in** **Figure 4D** **.** Upper panels: Cells derived from tumors of AC133.1-Alexa488-injected mice, but non-stained with the CD11b-PE-Cy7 antibody (Cy7/FL5 autofluorescence). Lower panel: Statistical analysis of the proportion of CD11b-positive cells having bound the injected AC133.1-Alexa488 antibody (n = 6). FL, fluorescence; WT, wild-type
(TIF)

Figure S6 **Determination of the lowest number of HCT116 wild-type cells detectable s.c.** ***in vivo*** **by FMT.** Increasing percentages of HCT116 wild-type cells were mixed with HCT116 p53−/− cells and stained with the mAb AC133-Cy5.5. The percentages of CD133-positive HCT116 wild-type and CD133-negative HCT116 p53−/− cells were confirmed by flow cytometry after staining with an anti-mouse PE-conjugated F(ab')_2_ fragment (middle panels); the single stain controls are shown in the upper panels. After mixing with matrigel, 2×10^6^ cells of these cell mixtures (in a total volume of 50 µl) were injected s.c. into the flanks of nude mice. At the lower left, representative 2D planar and 3D tomographic scans performed with an FMT1500 are shown. The injection sites of the cells are indicated by yellow arrowheads, on the 2D image along with the percentages of the CD133-positive HCT116 wild-type cells. In the 3D image, non-specific signals detected from the interior of the body are indicated by gray arrowheads. The panel at the lower right presents the amount of Cy5.5 fluorochrome at the individual injection sites (n = 6 animals). WT, wild-type
(TIF)
