# Supplementary figures and images for: Non-Invasive In Vivo Imaging of Tumor-Associated CD133/Prominin
Source: PLoS One. 2010 Dec 20;5(12):e15605. doi: 10.1371/journal.pone.0015605 (PMC3004948; doi:10.1371/journal.pone.0015605)

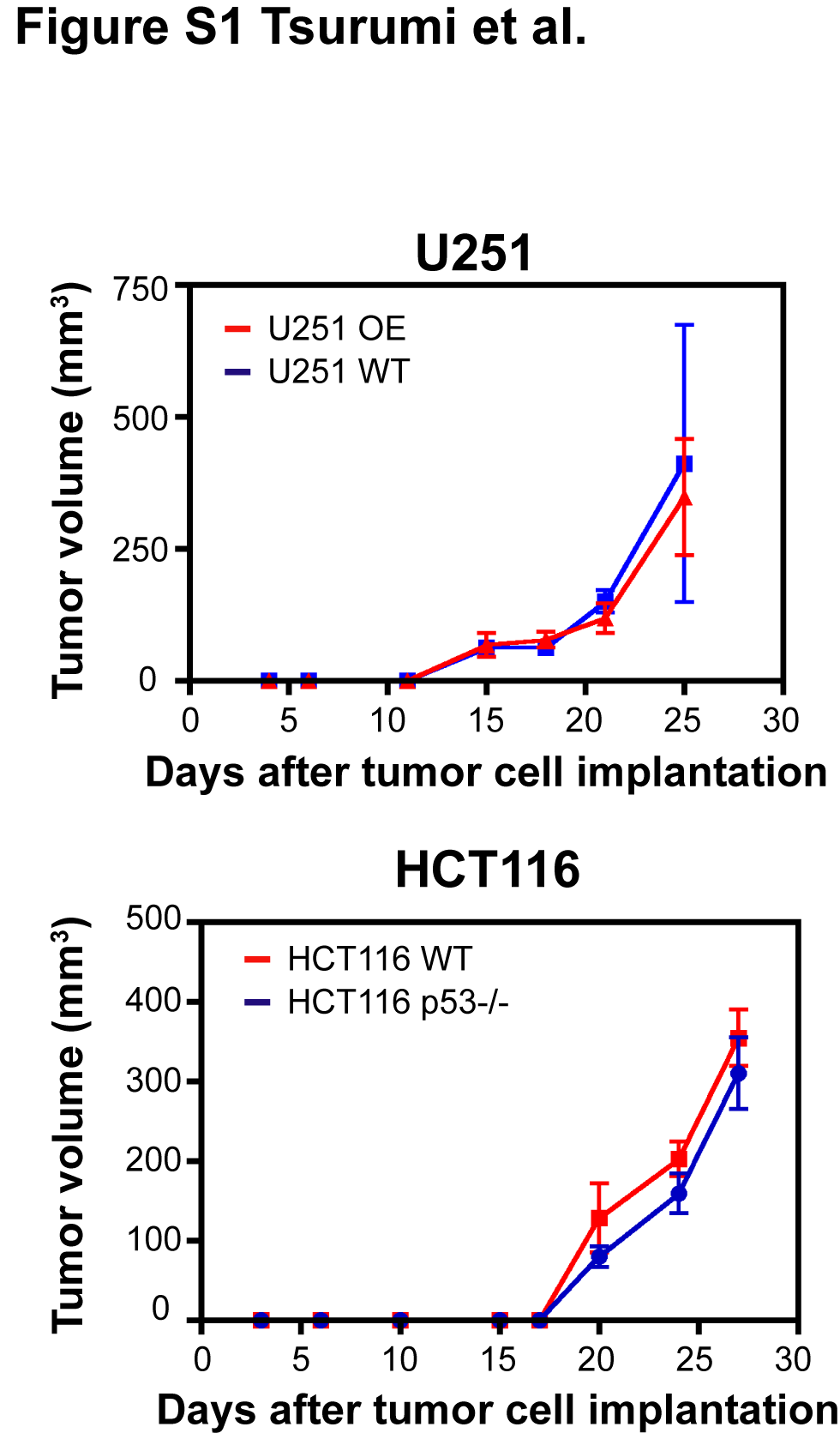

Supplement: Figure S1 — Growth curves for U251 and HCT116 xenograft tumors. Nude mice were injected s.c. with 5×10^6^ cells with the exception of HCT116 p53−/− cells which were injected at a number of 4×10^6^ to adjust the growth rate to the HCT116 wild-type tumors. Tumor sizes were measured twice weekly with a caliper. Individual tumor volumes were calculated by the formula V = [length × (width)2]/2. WT, wild-type; OE, CD133 overexpressing (TIF) [file pone.0015605.s001.tif]

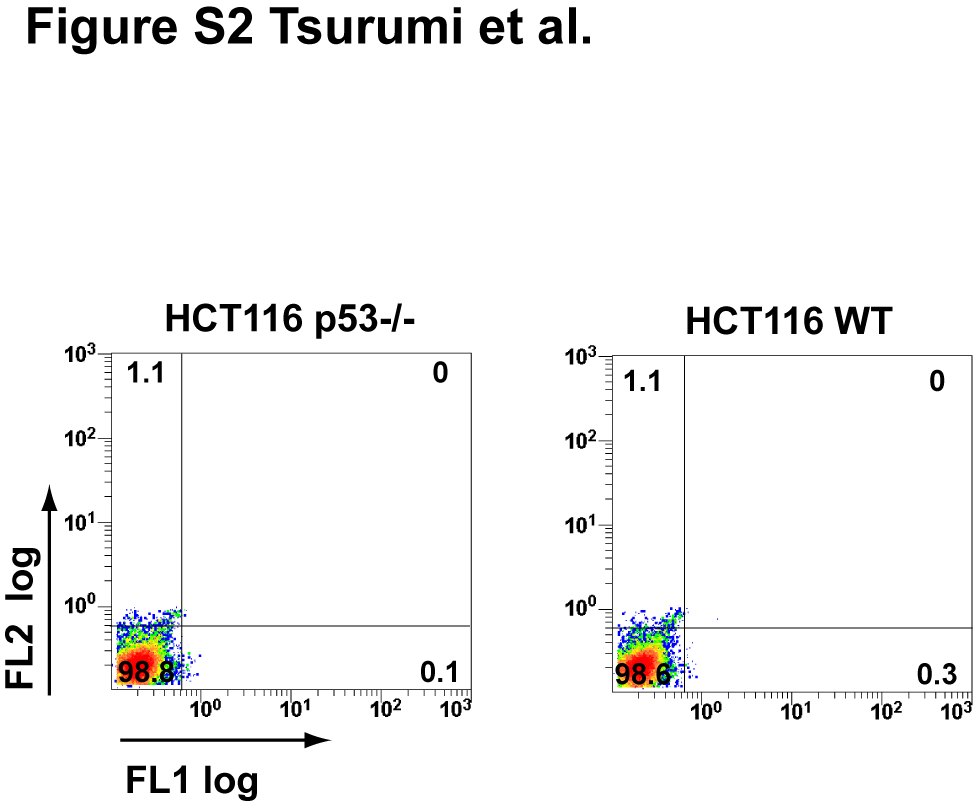

Supplement: Figure S2 — Autofluorescence of the cells used for the flow cytometric analyses presented in Figure 4A . The percentages of cells falling into the negative, single- and double-positive quadrants are shown. FL, fluorescence; WT, wild-type (TIF) [file pone.0015605.s002.tif]

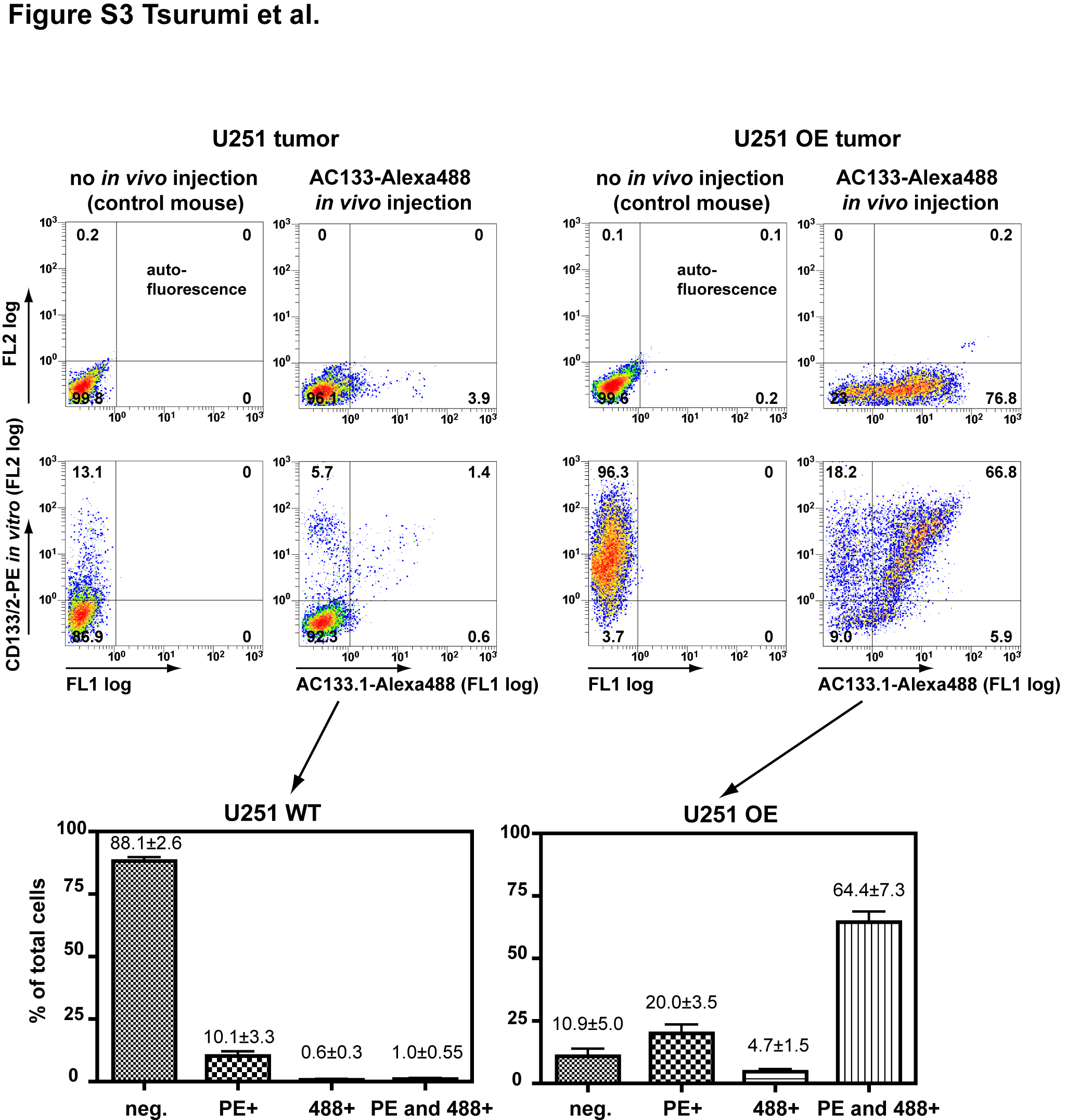

Supplement: Figure S3 — Controls and statistics for the flow cytometric analyses of the U251 tumors presented in Figure 4B . The upper panels demonstrate the gating according to the autofluorescence. Upper left subpanels: Autofluorescence of tumor cells from ‘control mice’ that had not been injected with AC133.1-Alexa488 antibody. Lower left subpanels: Fluorescence of tumor cells from non-injected ‘control mice’ stained with a PE-labeled CD133/2-specific antibody in vitro. Upper right subpanels: Fluorescence of tumor cells from mice that had been injected with AC133.1-Alexa488 antibody. Lower right subpanels: Fluorescence of tumor cells from injected mice stained with a PE-labeled CD133/2-specific antibody in vitro as shown in Figure 4B. The lower two panels show a statistical analysis of the percentages of tumor cells falling into the four quadrants (n = 3). FL, fluorescence; WT, wild-type; OE, overexpressing (TIF) [file pone.0015605.s003.tif]

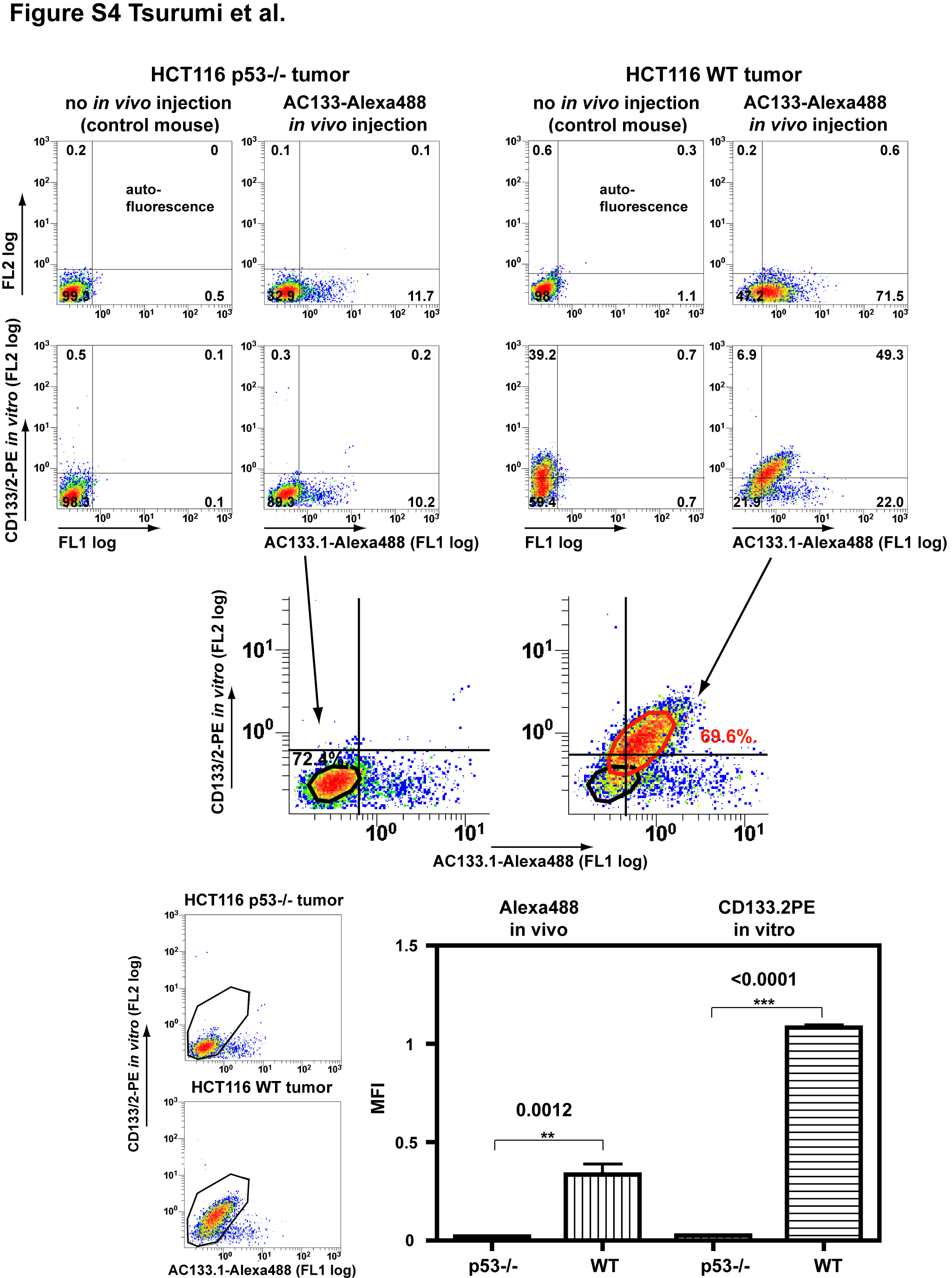

Supplement: Figure S4 — Controls and statistics for the flow cytometric analyses of the HCT116 tumors presented in Figure 4B . The upper panels demonstrate the gating according to the autofluorescence. Upper left subpanels: Autofluorescence of tumor cells from ‘control mice’ that had not been injected with AC133.1-Alexa488 antibody. Lower left subpanels: Fluorescence of tumor cells from non-injected ‘control mice’ stained with a PE-labeled CD133/2-specific antibody in vitro. Upper right subpanels: Fluorescence of tumor cells from mice that had been injected with AC133.1-Alexa488 antibody. Lower right subpanels: Fluorescence of tumor cells from injected mice stained with a PE-labeled CD133/2-specific antibody in vitro as shown in Figure 4B. The percentages of cells falling into the negative, single- and double-positive quadrants are shown. The middle and the lower panels show biological comparison controls (HCT116 wild-type vs. HCT116 p53−/− xenograft cells). Middle panel: The red and the black circled regions include the main cell population of a wild-type and a p53−/− xenograft, respectively. These populations do not overlap, demonstrating that the majority of the CD133+ cells in the wild-type xenografts had bound the injected AC133.1-Alexa488 antibody. Lower right panel: Statistical analysis of the mean fluorescence intensities for the Alexa488 and the PE signal (n = 6 wild-type and 5 p53−/− tumors, respectively). The cell populations analyzed fell into the gates shown in the lower left panel. FL, fluorescence; MFI, mean fluorescence intensity; WT, wild-type; OE, overexpressing (TIF) [file pone.0015605.s004.tif]

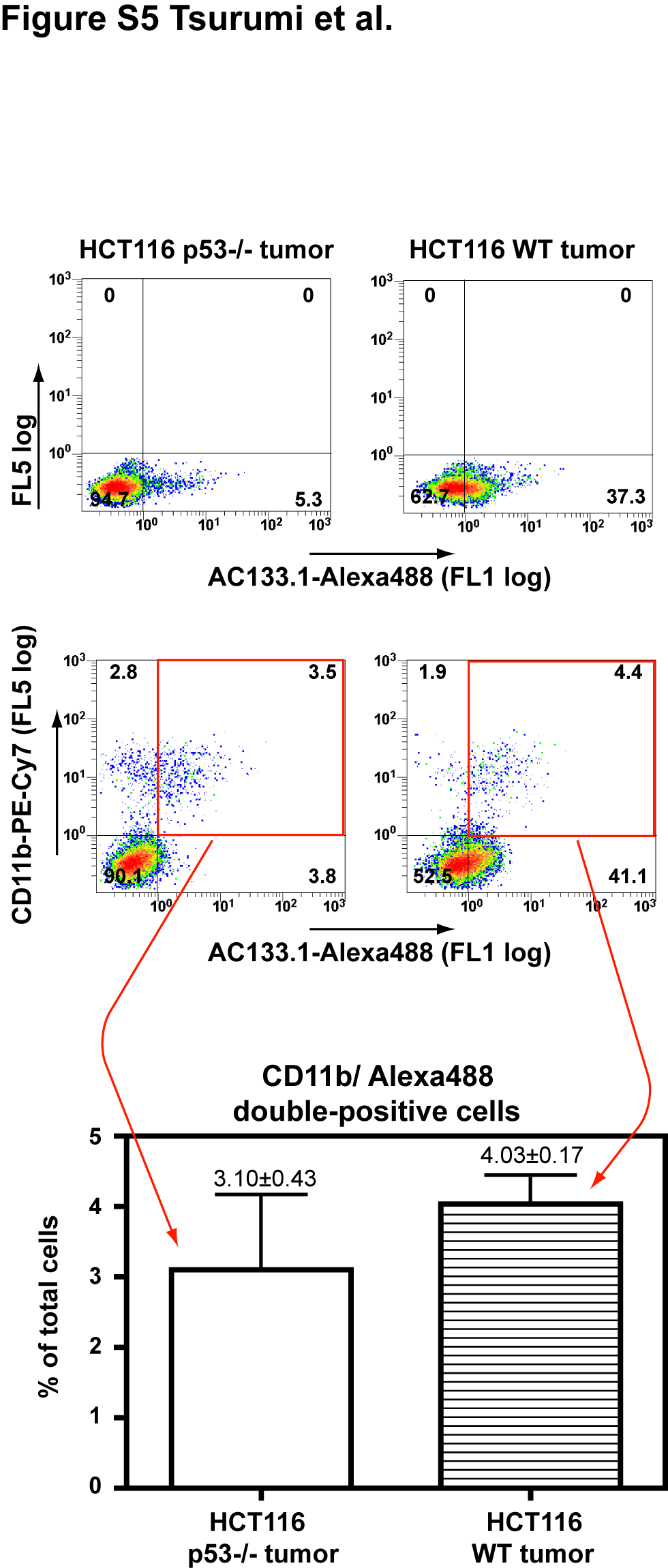

Supplement: Figure S5 — Controls and statistics for the flow cytometric analyses presented in Figure 4D . Upper panels: Cells derived from tumors of AC133.1-Alexa488-injected mice, but non-stained with the CD11b-PE-Cy7 antibody (Cy7/FL5 autofluorescence). Lower panel: Statistical analysis of the proportion of CD11b-positive cells having bound the injected AC133.1-Alexa488 antibody (n = 6). FL, fluorescence; WT, wild-type (TIF) [file pone.0015605.s005.tif]

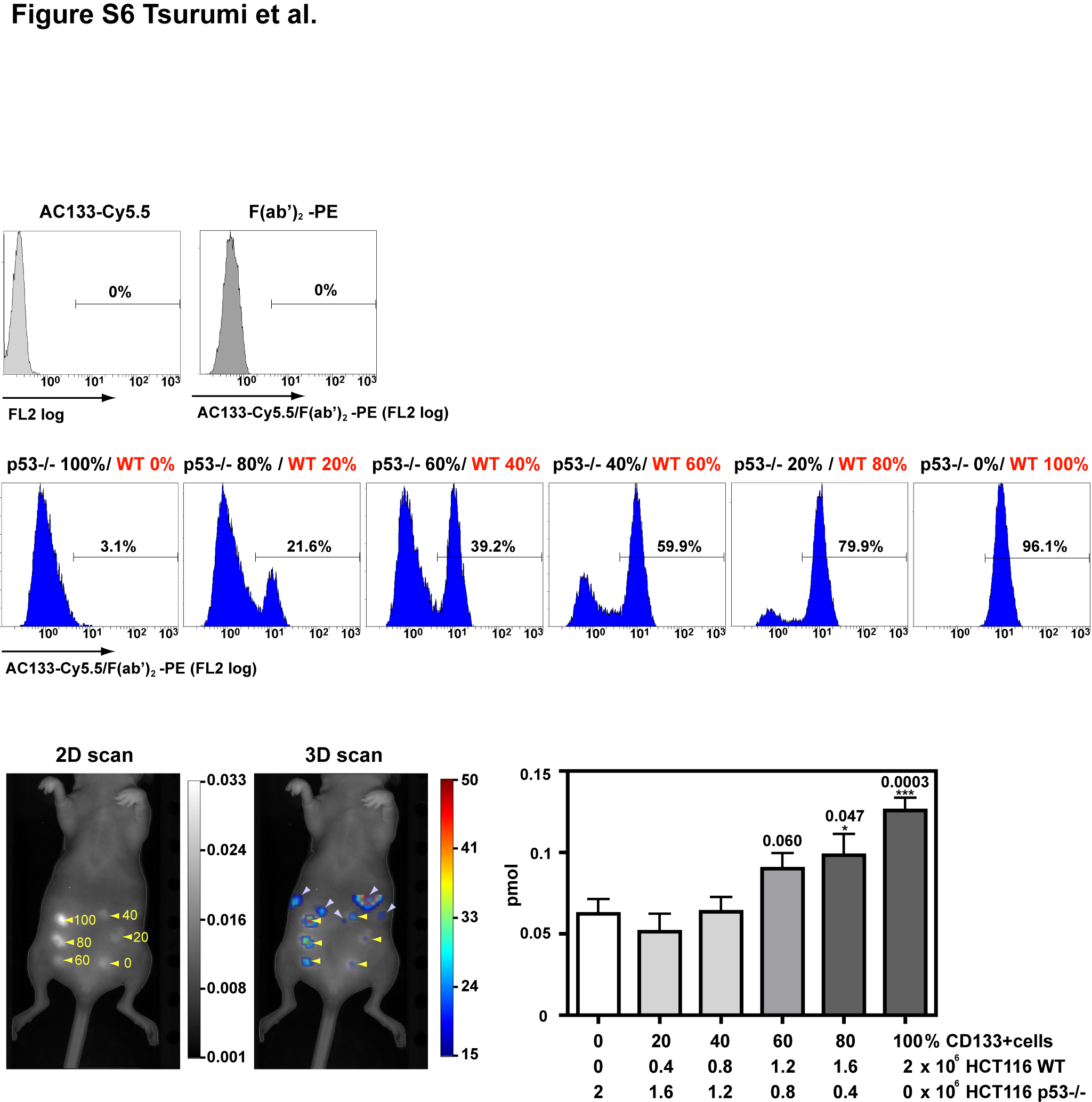

Supplement: Figure S6 — Determination of the lowest number of HCT116 wild-type cells detectable s.c. in vivo by FMT. Increasing percentages of HCT116 wild-type cells were mixed with HCT116 p53−/− cells and stained with the mAb AC133-Cy5.5. The percentages of CD133-positive HCT116 wild-type and CD133-negative HCT116 p53−/− cells were confirmed by flow cytometry after staining with an anti-mouse PE-conjugated F(ab')2 fragment (middle panels); the single stain controls are shown in the upper panels. After mixing with matrigel, 2×10^6^ cells of these cell mixtures (in a total volume of 50 µl) were injected s.c. into the flanks of nude mice. At the lower left, representative 2D planar and 3D tomographic scans performed with an FMT1500 are shown. The injection sites of the cells are indicated by yellow arrowheads, on the 2D image along with the percentages of the CD133-positive HCT116 wild-type cells. In the 3D image, non-specific signals detected from the interior of the body are indicated by gray arrowheads. The panel at the lower right presents the amount of Cy5.5 fluorochrome at the individual injection sites (n = 6 animals). WT, wild-type (TIF) [file pone.0015605.s006.tif]
